# Microcirculatory Dysfunction and Its Role in Diagnosing Acute Rejection in Pediatric Heart Transplantation: A Pilot Study

**DOI:** 10.3390/diagnostics15050545

**Published:** 2025-02-24

**Authors:** Borja Rivero-Santana, Enrique Balbacid-Domingo, César Abelleira-Pardeiro, Carlos Labrandero de Lera, Viviana Arreo del Val, Santiago Jiménez-Valero, María Fernández-Velasco, Raúl Moreno, Federico Gutiérrez-Larraya

**Affiliations:** 1 Cardiology Department, La Paz University Hospital, 28046 Madrid, Spain; 2 Hospital La Paz Institute for Health Research, IdiPAZ, 28046 Madrid, Spain; 3 Pediatrics Department, La Paz University Hospital, 28046 Madrid, Spain; 4 Investigación Biomédica en Red de Enfermedades Cardiovasculares (CIBER-CV), Instituto de Salud Carlos III, 28029 Madrid, Spain

**Keywords:** pediatric heart transplantation, microcirculation, acute rejection, coronary physiology, IMR, echocardiography

## Abstract

**Background/Objectives**: Acute rejection remains a major challenge in pediatric heart transplantation (HT), with limited tools for early diagnosis. In adult HT recipients, microcirculatory dysfunction, as measured by the index of microcirculatory resistance (IMR), has been identified as a potential biomarker of rejection. However, its role in pediatric populations is largely unexplored. This pilot study aimed to evaluate the association between coronary microcirculatory dysfunction and acute rejection in pediatric heart transplant recipients, as well as its relationship with echocardiographic alterations. **Methods**: This prospective, single-center study included 10 pediatric HT recipients who underwent routine coronary angiography and endomyocardial biopsy. The IMR, coronary flow reserve (CFR), and fractional flow reserve (FFR) were assessed. Acute rejection was classified as either acute cellular rejection (ACR) or antibody-mediated rejection (AMR) based on ISHLT criteria. Echocardiographic parameters included left ventricular ejection fraction (LVEF), global longitudinal strain (GLS), right ventricular (RV) dysfunction, and diastolic function. Patients were followed for a median of 9.7 months [IQR: 7.0–11.7]. **Results**: Patients with a history of acute rejection (40%, *n* = 4) were exclusively found in the IMR ≥ 15 group (66.7%), while no cases were observed in the IMR < 15 group (0%; *p* = 0.04). During follow-up, only one patient experienced acute rejection, occurring in the IMR ≥ 15 group, although the difference between groups was not statistically significant (*p* = 0.39). Both LVEF and GLS were worse in patients with IMR ≥ 15 compared to IMR < 15 (62.5% vs. 76.3% and −17.3% vs. −18.8%, respectively), although these differences did not reach statistical significance. No complications were reported during coronary physiology assessment. **Conclusions**: Microcirculatory dysfunction, as measured by IMR, was significantly associated with a history of acute cellular rejection in pediatric heart transplant recipients. While its predictive value for acute rejection during follow-up remains unclear due to the small sample size, this pilot study highlights the safety and feasibility of coronary physiology assessment in this population. Larger studies are needed to validate these findings and establish pediatric-specific diagnostic thresholds.

## 1. Introduction

Heart transplantation (HT) remains the definitive treatment for children with end-stage heart failure (HF), offering a critical chance of survival in an otherwise fatal condition [1,2]. Despite substantial advances in surgical techniques, immunosuppressive therapies, and post-transplant care, acute rejection (AR) continues to pose a major challenge, particularly in pediatric recipients. Both acute cellular rejection (ACR) and antibody-mediated rejection (AMR) are associated with significant morbidity and graft loss, yet early diagnosis remains elusive due to the lack of reliable biomarkers and the often subclinical nature of rejection [3,4]. Current diagnostic strategies, including endomyocardial biopsy (EMB), are invasive and have limitations in sensitivity and specificity, underscoring the need for alternative approaches to improve early detection and intervention [5].

In adult HT recipients, coronary microcirculatory dysfunction has emerged as a promising marker of rejection, with strong associations with graft failure and mortality [6]. The index of microcirculatory resistance (IMR) and coronary flow reserve (CFR) are established tools used to detect subclinical microvascular abnormalities that may precede overt rejection. These findings highlight the potential of microcirculatory assessment not only as a diagnostic tool but also as a means to identify graft dysfunction at an earlier stage [7,8]. However, evidence in pediatric populations is scarce, and the role of coronary microcirculation in this unique group remains poorly understood.

To date, no studies have systematically evaluated the relationship between microcirculatory dysfunction and rejection in pediatric HT recipients, leaving a critical gap in knowledge. Recognizing the need for age-specific diagnostic tools, we conducted this pilot study to investigate the association between coronary microcirculatory dysfunction and acute rejection in children after HT.

## 2. Materials and Methods

### 2.1. Study Design and Population

This prospective, single-center study was conducted at University Hospital La Paz (Madrid, Spain) between June 2023 and June 2024. Pediatric HT recipients with more than 1-year post transplantation and under 18 years of age undergoing routine follow-up coronary angiography and endomyocardial biopsy were consecutively enrolled. The study protocol was approved by the institutional review board, and written informed consent was obtained from the parents or legal guardians of all participants. This study was conducted in accordance with the principles of the Declaration of Helsinki.

Exclusion criteria included recent infection within 4 weeks prior to the procedure, hemodynamic instability (including cardiogenic shock or severe coagulopathy), second-degree or total atrioventricular block without pacemaker implantation, hemodynamically significant valvular disease, severe chronic obstructive pulmonary disease, pulmonary hypertension, bronchospasm, weight less than 30 kg, or any condition deemed unsuitable for an invasive procedure by the physician-in-charge. Additionally, patients with evidence of cardiac allograft vasculopathy (CAV) during the study angiography were excluded.

### 2.2. Physiological Assessment and Microcirculatory Dysfunction Definition

Coronary physiological assessment was performed using a 6F guide catheter without side holes and a pressure/temperature sensor guide wire (PressureWire™; Abbott Vascular Inc., Santa Clara, CA, USA) positioned in the mid-to-distal segment of the left anterior descending artery following the administration of intracoronary nitroglycerin (100–200 μg) and pressure equalization. Proximal aortic pressure (Pa), distal coronary pressure (Pd), and mean transit times were recorded under resting and hyperemic conditions. Hyperemia was induced via intravenous adenosine infusion at 140 μg/kg/min over 2–3 min, ensuring maximal hyperemia through hemodynamic changes such as increased heart rate or reduced mean arterial pressure. After completing the measurements, the guide wire was pulled back to the guide catheter to assess pressure drift. If a drift exceeding 0.12 FFR units was detected, re-equalization and repeated measurements were performed to ensure accuracy.

Coronary circulatory function was evaluated using three invasive physiological indices: fractional flow reserve (FFR), CFR, and the IMR. FFR, defined as the ratio of maximal coronary blood flow in a diseased artery to that in the same artery without stenosis, was utilized to assess the presence of hemodynamically significant epicardial stenosis. A value of FFR ≤ 0.80 was considered indicative of significant epicardial stenosis, while values > 0.80 were classified as normal. CFR, calculated as the ratio of hyperemic to baseline flow, evaluates the overall integrity of both epicardial and microvascular compartments and is particularly useful for assessing microvascular function when FFR is within the normal range. A CFR value < 2.0 was defined as abnormal, reflecting impaired coronary vasodilatory capacity [9]. IMR, derived from distal coronary pressure and hyperemic flow, represents a specific marker of microvascular resistance and was the primary metric used to assess coronary microvascular function. IMR, derived from distal coronary pressure and hyperemic flow, represents a specific marker of microvascular resistance and was the primary metric used to assess coronary microvascular function. IMR was calculated as the product of hyperemic Pd and hyperemic Tmn (IMR = Pd × Tmn). Microvascular dysfunction was defined as an IMR ≥ 15, according to the largest and most recent study conducted in adult heart transplant recipients [10].

### 2.3. Echocardiographic Assessment

Echocardiographic examinations were performed by experienced cardiologists trained in advanced echocardiography and familiar with the study, using a Philips EPIQ 7C (Philips Medical Systems, Andover, MA, USA). Left ventricular (LV) systolic function was assessed using left ventricular ejection fraction (LVEF), calculated by the biplane Simpson’s method, with values ≤50% considered abnormal. Global longitudinal strain (GLS) was measured using speckle-tracking echocardiography with Philips QLAB 10.2 software, with values greater than −18% indicating systolic dysfunction. Diastolic function was evaluated using pulsed-wave Doppler and tissue Doppler imaging at the mitral valve, assessing the E/A ratio (<0.8 suggesting impaired relaxation; >2.1 with a deceleration time <120 ms indicating a restrictive filling pattern) and the E/e’ ratio (>14 suggesting elevated LV filling pressures). Right ventricular (RV) dysfunction was defined as a fractional area change (FAC) <35%. Additionally, tricuspid annular plane systolic excursion (TAPSE) and the S wave were measured. TAPSE was assessed using M-mode echocardiography at the tricuspid annulus in the apical four-chamber view, while the S wave was measured using tissue Doppler imaging (TDI) at the tricuspid annulus.

### 2.4. Acute Rejection Definitions and Protocol for the Diagnosis

Acute rejection was classified as acute cellular rejection (ACR) or antibody-mediated rejection (AMR) based on the International Society for Heart and Lung Transplantation (ISHLT) criteria [11]. ACR was graded as Grade 0R, indicating no evidence of rejection; Grade 1R, characterized by mild rejection with interstitial and/or perivascular lymphocytic infiltrates and up to one focus of myocyte damage; Grade 2R, indicating moderate rejection with two or more foci of lymphocytic infiltration and associated myocyte damage; and Grade 3R, reflecting severe rejection with diffuse infiltrates, multifocal myocyte damage, and additional findings such as edema, hemorrhage, or vasculitis. AMR was diagnosed using a combination of histopathological and immunohistochemical findings, including evidence of capillary damage, macrophage infiltration, interstitial edema, and intravascular thrombi, along with complement deposition such as C4d or C3d and intravascular immunoglobulins (IgG, IgA, IgM). The severity of AMR was categorized as pAMR 0, with no evidence of rejection; pAMR 1 (H+), with histological abnormalities only; pAMR 1 (I+), with immunopathological abnormalities only; pAMR 2, with combined histological and immunopathological findings; and pAMR 3, with severe rejection characterized by extensive endothelial injury, capillary damage, and interstitial hemorrhage.

EMBs were performed via femoral venous access following standard procedural protocols. Tissue samples were analyzed using hematoxylin–eosin staining to assess microvasculopathy, including endothelial swelling and vessel wall thickening, while circulating antibodies such as anti-HLA class I/II and anti-MICA were measured to complement the biopsy findings. All samples were evaluated by a pathologist blinded to microcirculatory data to ensure diagnostic accuracy and adherence to international standards.

### 2.5. Patient Follow-Up

Patients were followed for a minimum of six months after their initial assessment. During this period, routine clinical evaluations, imaging studies, and laboratory tests were performed according to standard post-transplantation protocols to monitor for AR and manage it in line with established clinical guidelines. This six-month follow-up allowed for a comprehensive assessment of transplant outcomes and provided insights into the long-term impact of microcirculatory function on graft health.

### 2.6. Endpoints

The primary endpoints of this study were the association between the IMR and (1) a history of prior acute rejection, classified as either ACR or AMR, and (2) the development of new episodes of ACR or AMR during the current study or follow-up.

Secondary endpoints included (1) the association between IMR and echocardiographic abnormalities, specifically left ventricular ejection fraction (LVEF), global longitudinal strain (GLS), and right ventricular dysfunction, and (2) the safety of invasive coronary physiological assessment, evaluated by the occurrence of procedural complications such as coronary dissection, coronary spasm, arrhythmias (e.g., atrioventricular block), or hemodynamic instability during adenosine administration.

### 2.7. Statistical Analysis

Continuous variables were expressed as mean ± standard deviation (SD) or median with interquartile range (IQR), as appropriate, based on their distribution. Categorical variables were presented as frequencies and percentages. The Shapiro–Wilk test was used to assess the normality of continuous variables. Comparisons between groups were performed using the Student’s *t*-test or Mann–Whitney U test for continuous variables and the chi-square test or Fisher’s exact test for categorical variables, as applicable. Statistical significance was defined as a two-sided *p* value <0.05. All analyses were conducted using R statistical software (v4.2.2; R Core Team, 2022).

## 3. Results

### 3.1. Baseline Characteristics and Echocardiographic Assessment

Ten pediatric HT recipients were prospectively enrolled in this study. All patients underwent heart transplantation between 2010 and 2023. These baseline characteristics are summarized in Table 1. The primary indication for transplantation was dilated cardiomyopathy in 40% of the cases. The median age of the recipients was 12.8 ± 2.4 years, and the median time since transplantation was 5 years [IQR 2.5 to 6.7].

Echocardiographic assessment showed that 10% of patients presented with left ventricular systolic dysfunction, while 30% had alterations in GLS. Furthermore, 30% of patients exhibited right ventricular dysfunction. These echocardiographic findings are detailed in Table 2.

### 3.2. Acute Celular and Acute Antibody-Mediated Rejection

ACR had previously been documented in four patients (40% of the sample), all classified as grade 2R according to ISHLT criteria. However, during the current study focusing on microcirculatory assessment, ACR was observed in only one patient, also classified as grade 2R. Samples for biopsy and laboratory analysis were obtained from all patients during the catheterization procedure. In contrast, AMR was not detected in any patient at any point. Furthermore, there was no evidence of circulating anti-HLA class I, class II, or MICA antibodies in the collected samples.

### 3.3. Coronary Microcirculatory Dysfunction

Microcirculatory function assessment was successfully performed in all patients without any procedural complications. The mean FFR was 0.95 ± 0.03, confirming the absence of significant epicardial coronary artery disease. The median CFR was 2.4 ± 1.7, with abnormalities detected in 50% of patients. The mean IMR was 18.3 ± 8.6, with elevated values (IMR ≥ 15) observed in 60% of the cohort. These findings are summarized in Table 2.

### 3.4. Study Endpoints

#### 3.4.1. Primary Endpoints

A. IMR in Relation to Previous Acute Rejection

**Figure 1:**
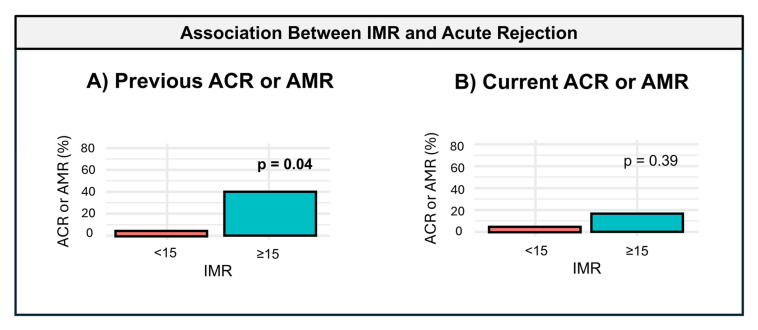
Association between IMR and acute rejection. The association between the index of microcirculatory resistance (IMR) and acute rejection episodes is depicted. IMR categories (<15 and ≥15) are compared for the prevalence of acute rejection, including acute cellular rejection (ACR) and antibody-mediated rejection (AMR): (**A**) prevalence of previous rejection episodes (ACR or AMR), showing a significant association (*p* = 0.04); (**B**) prevalence of current rejection episodes (ACR or AMR), with no significant association observed (*p* = 0.39). The black horizontal lines within the boxes represent the median, and the boxes illustrate the interquartile range (IQR). Statistical significance of group differences is indicated by the *p* values. All detected rejection episodes were of the ACR type.

Patients with a history of AR (40%, *n* = 4) were exclusively found in the group with IMR > 15 (66.7%), while no cases of rejection were observed in the IMR < 15 group. This difference was statistically significant (*p* = 0.04). All cases of rejection were classified as grade 2R ACR, with no instances of AMR observed in the cohort. These findings are illustrated in Table 2 and Figure 1A.

B. IMR in Relation to Acute Rejection During Follow-up

The median follow-up period was 9.7 months [IQR: 7.0–11.7]. During this time, only one patient experienced AR, occurring at the time of the coronary study. This case was observed in the IMR ≥ 15 group, with no episodes in the IMR < 15 group. Although AR was exclusively identified in the IMR > 15 group, the difference between groups was not statistically significant (*p* = 0.39). The rejection was classified as grade 2R ACR, with no cases of AMR reported. These findings are illustrated in Table 2 and Figure 1B.

#### 3.4.2. Secondary Endpoints

A. IMR in Relation to Echocardiographic Assessment

**Figure 2:**
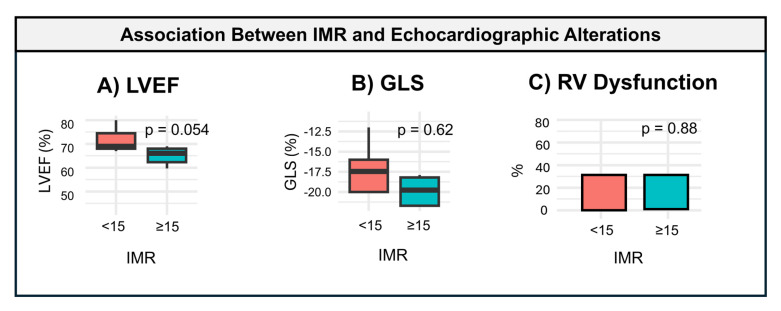
Association between IMR and echocardiographic alterations. The association between the index of microcirculatory resistance (IMR) and echocardiographic parameters is depicted. IMR categories (<15 and ≥15) are compared for the following: (**A**) left ventricular ejection fraction (LVEF), showing a borderline significance (*p* = 0.054); (**B**) global longitudinal strain (GLS), with no significant differences (*p* = 0.62); (**C**) right ventricular (RV) dysfunction, also showing no significant differences (*p* = 0.88). The black horizontal lines within the boxes represent the median, and the boxes illustrate the interquartile range (IQR). Statistical significance is indicated by the *p* values.

Echocardiographic parameters, including left ventricular ejection fraction (LVEF), global longitudinal strain (GLS), right ventricular dysfunction, and diastolic function, were evaluated in relation to microcirculatory dysfunction. Both LVEF and GLS were worse in patients with an IMR ≥ 15 compared to those with an IMR < 15 (62.5% vs. 76.3% and −17.3% vs. −18.8%, respectively). However, these differences did not reach statistical significance (*p* = 0.054 and *p* = 0.622, respectively). In contrast, no significant differences were observed in right ventricular dysfunction or diastolic function between the two groups. These findings are illustrated in Figure 2.

B. Safety Endpoint

No complications occurred during the coronary physiology procedure. Specifically, there were no cases of coronary dissection, coronary spasm, arrhythmias (such as atrioventricular block), or any other procedure-related adverse events.

## 4. Discussion

This study is the first to evaluate the use of coronary physiology, specifically the IMR, to investigate its association with AR in pediatric HT recipients. Our findings demonstrate that IMR ≥ 15 is significantly associated with a history of AR, with no rejection observed in patients with IMR < 15. Additionally, echocardiographic parameters such as LEVF and GLS were worse in patients with elevated IMR values, although these differences did not reach statistical significance. Importantly, this study highlights the feasibility and safety of invasive coronary physiological assessments in this vulnerable population, with no procedural complications reported.

### 4.1. Challenges in Diagnosing Acute Rejection

AR remains a significant complication in pediatric HT, contributing to graft failure and increased morbidity [12,13]. ACR is the most common form, affecting up to 40% of pediatric recipients during follow-up [14]. AMR, though less frequent, is particularly challenging due to the unclear pathogenic implications of donor-specific antibodies (DSAs), the lack of reliable biomarkers for early detection, and the low sensitivity of findings in endomyocardial biopsy [4,15]. Current diagnostic methods rely heavily on EMB, which, despite being the gold standard, is invasive and prone to sampling errors [16]. Up to 5% of severe rejection episodes are only identified after the development of hemodynamic instability, underscoring the urgent need for less invasive and more sensitive diagnostic tools [17].

In this context, coronary microcirculatory dysfunction, as assessed by IMR, has emerged as a promising functional biomarker. A landmark study by Joo Myung Lee et al. [10] demonstrated that IMR ≥ 15 measured one month after HT in adults was strongly associated with a 15-fold increased risk of biopsy-proven ACR of grade ≥2R over two years of follow-up. Their findings highlighted the incremental prognostic value of IMR over clinical variables, significantly improving risk stratification for ACR. Our results align with these findings but extend the evidence to a pediatric population. However, our study differs in focusing on a stable post-transplant cohort beyond the acute phase, where transient hemodynamic changes are less likely to confound IMR measurements. This distinction underscores the potential of IMR as a long-term indicator of graft health in children.

### 4.2. Echocardiographic Alterations

In our study, LVEF and GLS tended to be worse in patients with IMR ≥ 15, but these differences did not reach statistical significance. While this may suggest a link between microvascular dysfunction and graft function, it is unclear whether these echocardiographic changes are a direct consequence of elevated IMR, reflect prior rejection episodes, or are coincidental findings.

### 4.3. Persistent Microvascular Dysfunction

The significant association between IMR ≥ 15 and a history of AR observed in our study suggests that microvascular dysfunction may persist even after rejection has been treated. Despite advances in immunosuppressive therapies, rejection episodes appear to induce lasting damage to the coronary microvasculature, potentially increasing graft vulnerability. This highlights the need for therapeutic strategies to restore microvascular integrity and prevent chronic graft injury. Additionally, the single case of ACR observed during follow-up reinforces the potential utility of IMR in predicting rejection risk. Longitudinal studies are essential to determine whether IMR normalizes, remains stable, or worsens in patients with recurrent rejection. Understanding these trajectories could inform individualized post-transplant care and improve long-term outcomes.

### 4.4. Clinical Implications

While this study does not establish IMR as a routine diagnostic tool, it underscores its potential utility as a complementary measure in post-transplant evaluations. Patients with elevated IMR values might benefit from closer monitoring and tailored follow-up strategies to better understand the impact of microvascular dysfunction on long-term outcomes. The results suggest the need to adapt IMR measurement to the pediatric population using a larger sample, allowing its potential implementation in clinical practice for the early detection and prevention of transplant rejection, ultimately improving outcomes in pediatric HT recipients.

Future studies should focus on validating IMR thresholds specific to pediatric patients and evaluating its sensitivity and specificity in detecting early-stage rejection, thereby enabling its potential integration into clinical practice. If future studies confirm the ability of IMR to accurately determine rejection risk, current protocols regarding the role of endomyocardial biopsy—which remains crucial for post-transplant management—may need to be reconsidered. However, before IMR can be integrated into routine practice, it is essential to evaluate its cost-effectiveness and determine whether a protocol shift would provide tangible clinical benefits. Addressing these questions in larger prospective studies will be crucial to assess the feasibility of incorporating IMR into post-transplant care.

### 4.5. Safety and Methodology

The safety and feasibility of coronary physiology assessments in pediatric HT recipients were clearly demonstrated in this study. By implementing a standardized protocol—including adenosine infusion at 140 μg/kg/min to induce hyperemia and the use of specialized pressure/temperature sensor guide wires—we successfully performed IMR measurements without complications. No events of coronary dissection, spasm, arrhythmias, or hemodynamic instability were observed. These findings establish a reproducible framework for future research and clinical application, paving the way for broader integration of coronary physiology assessments in routine post-transplant care.

### 4.6. IMR vs. CFR in Assessing Microcirculatory Dysfunction

In our study, all patients had normal fractional flow reserve (FFR) values, confirming the absence of significant epicardial coronary artery disease. IMR was used to define microcirculatory dysfunction, as it provides a specific measure of microvascular resistance during maximal hyperemia. While CFR generally aligned with IMR in our cohort, one discordant case illustrated its limitations. CFR reflects the combined function of epicardial and microvascular compartments and can be influenced by hemodynamic factors such as heart rate or baseline coronary flow, potentially masking true microvascular dysfunction, as highlighted in other contexts [18]. Conversely, IMR is unaffected by baseline conditions, offering a more robust and specific evaluation of microvascular health. Although the reliability of IMR is well established, future studies could explore the combined use of IMR and CFR to enhance diagnostic accuracy and potentially offer additional insights into graft health.

### 4.7. Implications for AMR

While AMR was not observed in our cohort, its association with microvascular dysfunction warrants further investigation. Current diagnostic markers for AMR, such as complement deposition and DSAs, are limited in sensitivity and specificity [19,20]. If it can be demonstrated in a larger cohort of patients that IMR detects microvascular changes before histopathological findings emerge, this could provide an opportunity for earlier therapeutic intervention, such as the timely administration of complement inhibitors like eculizumab [21]. Such an approach may help mitigate the progression of microvascular injury and improve outcomes for pediatric HT recipients.

### 4.8. Future Directions

Future research should aim to establish whether IMR can reliably and at an early stage detect both cellular and antibody-mediated rejection in pediatric populations. This includes validating IMR thresholds specific to children and evaluating its sensitivity and specificity in detecting early-stage rejection. Additionally, integrating IMR with current diagnostic techniques, such as DSAs, endomyocardial biopsy, and advanced imaging modalities, could enhance diagnostic precision and enable earlier therapeutic interventions. Combining these tools might offer a pathway to optimized rejection management and tailored treatment strategies. Further studies should also investigate the potential role of IMR in guiding therapeutic decisions, monitoring treatment efficacy, and predicting long-term outcomes. By advancing these efforts, we could transition toward less invasive yet highly effective diagnostic and monitoring protocols, ultimately improving the prognosis and care of pediatric HT recipients.

### 4.9. Limitations

This study has several limitations. Firstly, the small sample size reduces statistical power and limits the generalizability of the findings. Nevertheless, as a pilot study, it was designed to evaluate the feasibility of microcirculatory assessment in pediatric HT recipients and to explore its potential role in rejection diagnosis. The complexity of pediatric HT and challenges in obtaining informed consent precluded a larger sample size at this stage, but these results lay the groundwork for future, larger-scale studies. Secondly, the observational and single-center design may introduce selection and procedural biases, potentially affecting the external validity of the findings. Multicenter studies are essential to confirm the applicability of these results in diverse clinical settings. Thirdly, the absence of AMR cases in our cohort limited our ability to investigate the relationship between microcirculatory dysfunction and this specific rejection type. Early diagnosis of AMR in pediatric populations remains a challenge and warrants further exploration. Finally, while the findings are promising, they do not establish microcirculatory dysfunction as a routine diagnostic tool in clinical practice. Further multicenter studies with larger cohorts are required to validate these results, refine pediatric-specific diagnostic thresholds, and better characterize the relationship between microcirculatory dysfunction and both ACR and AMR.

## 5. Conclusions

This study identifies a significant association between microcirculatory dysfunction, as measured by IMR, and a history of acute rejection in pediatric HT recipients. While its predictive value for early rejection could not be demonstrated due to the pilot nature and small sample size, IMR holds promise as a diagnostic tool. The high prevalence of microcirculatory changes underscores the need for tailored assessment methods in pediatric patients. This study also confirms the safety and feasibility of invasive coronary physiological assessment, with no complications, reinforcing its potential for integration into clinical practice. Larger studies are needed to confirm these findings and establish pediatric-specific IMR thresholds.

## Figures and Tables

**Table 1:** Baseline clinical characteristics according to IMR value.

	IMR < 15 (*n* = 4)	IMR ≥ 15 (*n* = 6)	Total (*n* = 10)	*p* Value
Demographics				
Age, years	12.2 (2.1)	13.2 (2.7)	12.8 (2.4)	0.584
Female	3 (75%)	1 (16.7%)	4 (4%)	0.065
HT indication				0.076
DCM	1 (25%)	3 (50%)	4(40%)	
NCCM	N/A	3 (5%)	3 (30%)	
RCM	2 (50%)	N/A	2 (20%)	
Shone Complex	1 (25%)	N/A	1 (10%)	
BMI, kg/m^2^	21.2 (4.5)	18.7 (5.7)	19.7 (5.1)	0.477
Height, cm	156.3 (16.2)	153.4 (13.0)	154.2 (13.1)	0.763
Weight, kg	59.0 (0.9)	46.8 (13.4)	50.5 (12.5)	0.165
Post transplant, years	3.9 [3.5; 4.9]	6.6 [2.2; 8.7]	4.2 [3.2; 8.4]	0.402

Values are mean ± SD, median [IQR], or *n* (%). IMR indicates index of microcirculatory resistance. Data are presented by IMR categories (<15 vs. ≥15). *p* values in bold indicate statistical significance. Abbreviations: AR: acute rejection; DCM: dilated cardiomyopathy; N/A: no events recorded; NCCM: non-compaction cardiomyopathy; RCM: restrictive cardiomyopathy.

**Table 2:** Baseline clinical characteristics according to IMR value.

	IMR < 15 (*n* = 4)	IMR ≥ 15 (*n* = 6)	Total (*n* = 10)	*p* Value
Echocardiographic assessment				
LV				
LVEF, %	76.3 (6.4)	62.5 (9.6)	66.7 (10.7)	0.054
GLS, %	−18.8 (3.9)	−17.3 (4.6)	−18.4 (3.9)	0.622
E/A	2.8 (1.2)	2.3 (0.7)	2.5 (0.9)	0.394
E/e’	8.4 (3.1)	6.9 (1.2)	7.5 (2.2)	0.307
RV				
TAPSE, mm	14.8 (3.2)	13.1 (4.0)	13.8 (3.6)	0.508
S wave, cm/s	9.4 (2.8)	9.3 (1.2)	9.3 (1.8)	0.888
RV dysfunction	1.0 (33.3%)	2.0 (33.3%)	3.0 (30.0%)	0.880
Physiological indices				
FFR	0.96 (0.02)	0.94 (0.03)	0.95 (0.03)	0.357
CFR	4.6 (2.3)	1.8 (1.1)	2.4 (1.7)	**0.022**
IMR	10.2 (5.6)	23.7 (5.2)	18.3 (8.6)	**0.005**

Values are mean ± SD, median [IQR], or *n* (%). IMR indicates index of microcirculatory resistance. Data are presented by IMR categories (<15 vs. ≥15). Abbreviations: CFR: coronary flow reserve; GLS: global longitudinal strain; LV: left ventricle; LVEF: left ventricular ejection fraction; RV: right ventricle; TAPSE: tricuspid annular plane systolic excursion. RV dysfunction was defined as a fractional area change (FAC) <35%.

## Data Availability

The data supporting the reported results are available upon request from the corresponding author. Due to privacy and ethical restrictions, the datasets are not publicly accessible.

## Abbreviations

The following abbreviations are used in this manuscript.

AR	Acute rejection
ACR	Acute cellular rejection
AMR	Antibody-mediated rejection
CFR	Coronary flow reserve
GLS	Global longitudinal strain
HT	Heart transplantation
IMR	Index of microcirculatory resistance
LVEF	Left ventricular ejection fraction
RV	Right ventricle
TAPSE	Tricuspid annular plane systolic excursion
